# Bacterial distribution on the ocular surface of patients with primary Sjögren’s syndrome

**DOI:** 10.1038/s41598-022-05625-w

**Published:** 2022-02-02

**Authors:** Yong Chan Kim, Baknoon Ham, Kui Dong Kang, Jun Myeong Yun, Man Jae Kwon, Hyun Seung Kim, Hyung Bin Hwang

**Affiliations:** 1 Department of Ophthalmology, Incheon St. Mary’s Hospital, College of Medicine, The Catholic University of Korea, #56 Dongsu-ro, Incheon, 21431 Republic of Korea; 2 Department of Earth and Environmental Sciences, Korea University, Seoul, Republic of Korea; 3 Department of Ophthalmology, Seoul St. Mary’s Hospital, College of Medicine, The Catholic University of Korea, Seoul, Republic of Korea

**Keywords:** Microbiome, Eye diseases, Medical research

## Abstract

Many studies have shown that gut microbial dysbiosis is a major factor in the etiology of autoimmune diseases but none have suggested that the ocular surface (OS) microbiome is associated with Sjögren’s syndrome (SS). In this prospective study, we analyzed bacterial distribution on the OS in patients with primary SS. Among the 120 subjects included in this study, 48 patients (group A) had primary SS, whereas 72 subjects (group B) had dry eye symptoms that were unrelated to SS. We evaluated clinical dry eye parameters such as the OS disease index, ocular staining score (OSS), Schirmer’s I test, and tear break-up time (TBUT). Conjunctival swabs were used to analyze the microbial communities from the two groups. Bacterial 16S rRNA genes were sequenced using the Illumina MiSeq platform, and the data were analyzed using the QIIME 1.9.1 program. The Shannon index was significantly lower in group A than in group B microbiota (p < 0.05). An analysis of similarity using the Bray–Curtis distance method found no difference in beta-diversity between the two groups (p > 0.05). In group A, *Actinobacteria* at the phylum level and *Corynebacteria* at the genus level exhibited low abundance than group B, but the differences were not statistically significant (p > 0.05). SS apparently decreases the diversity of the OS microbial community. These observations may be related to the pathophysiology of SS and should be investigated in future studies.

## Introduction

Sjögren’s syndrome (SS) is an autoimmune disease that is characterized by lymphocytic infiltration of exocrine glands such as salivary and lacrimal glands, resulting in dry eye disease and dry mouth^1^. Patients with SS and dry eyes exhibit particular glandular and mucosal immunopathological changes. CD4^+^ T cells and dendritic cells infiltrate conjunctival tissue, and inflammatory cytokines such as interferon gamma and interleukin (IL)-17, secreted from these T cells, induce apoptosis of epithelial and goblet cells, exacerbating dryness of the ocular surface (OS)^2,3^. In addition, CD8^+^ T cells are known to play an important role in the pathophysiology of SS, such as salivary gland damage^4^. Therefore, SS changes the immunological characteristics of the corneal and conjunctival surfaces.

Commensal microorganisms are present on the uninfected human OS, and several studies have used traditional microbiological culture techniques to study the microbiota of the OS^5–7^. Furthermore, deep sequencing of bacterial DNA has shown that various species of commensal microorganisms are present on the OS^8^.

However, it is unclear whether the species distribution of commensal microorganisms on the OS is altered in patients with SS. Unusual microbial flora distribution patterns are associated with some immunological diseases, and normalizing these distribution patterns is one approach to treatment. Crohn’s disease is a chronic inflammatory disorder of the gastrointestinal tract that results from an excessive innate and adaptive immune response to environmental factors in genetically susceptible subjects^9^. Crohn’s disease suppresses the function of regulatory T cells, which play an important role in immunological homeostasis^10^. Importantly, bacterial dysbiosis may be an important pathological consequence of Crohn’s disease, and restoring the microbial balance is one method of treating the disease^11,12^. In fact, patients with Crohn’s disease have more pathogenic bacterial species and fewer normal commensal bacterial species^13^. Zaheer et al. attempted to demonstrate an association between gut dysbiosis and SS in a CD25 knockout mouse model. The study revealed that lack of commensal gut bacteria aggravated dacryoadenitis and generated autoreactive CD4^+^ T cells, increasing pathogenicity in the knockout mice. Therefore, in this SS mouse model, the commensal gut bacteria and their metabolites may have an immunoregulatory function that protects the exocrine glands^14^.

However, it is difficult to establish a clear link between gut dysbiosis and pathology of the OS in patients with SS. Therefore, it is necessary to study the OS microbiota. Unfortunately, as Zaheer et al. reported, OS microbiota are sparse and difficult to analyze. Therefore, we used next-generation sequencing (NGS) to analyze numerous microbial populations distributed across the OS, similar to a previous study^15^. Here, we assess the composition of the OS microbial communities and evaluate the significance of the diversity and abundance of bacterial strains.

## Results

### Basic characteristics and dry eye parameters

Of the 120 subjects included in this study, 48 subjects (group A) were diagnosed with SS according to American College of Rheumatology/European League Against Rheumatism diagnostic criteria; the remaining 72 subjects (group B) did not have SS. Basic characteristics of all subjects are described in Table 1. In both groups, there were more females than males. Kolmogorov–Smirnov tests were used to confirm that the data were normally distributed (*p* > 0.05). The mean ages of groups A and B were 51.71 ± 9.46 years and 54.50 ± 13.60 years, respectively, and these ages were not significantly different (independent *t*-test, *p* > 0.05). There were no significant differences between the two groups in terms of clinical parameters for dry eyes such as ocular staining score (OSS), OS disease index (OSDI), tear break-up time (TBUT), and Schirmer’s test (independent *t*-tests, all *p* > 0.05).

**Table 1:** Ages and dry eye clinical parameters of the two groups.

	Age (years)	OSDI	TBUT (sec)	Schirmer value (mm)	OSS
Group A (M:F = 11:37))	51.71 ± 9.46	36.53 ± 25.24	4.90 ± 2.04	7.88 ± 7.67	3.10 ± 1.88
Group B (M:F = 4:68)	54.50 ± 13.60	41.94 ± 24.48	4.92 ± 1.77	8.43 ± 8.08	3.40 ± 2.02
p value	0.235	0.244	0.953	0.707	0.417

Independent t-test.

*OSDI* Ocular Surface Disease Index, *TBUT* tear break up time, *OSS* ocular staining score.

### Sequencing data

The 16S rRNA V4 amplicon libraries were sequenced from 120 individual samples. We obtained a total of 1,890,438 high-quality reads, and the number of observations (distinct operational taxonomic units [OTUs]) was 10,668. There was a mean ± standard deviation (SD) of 15,753.7 ± 6354.1 sequences per sample, with a median of 15,928 reads (range, 5001 to 37,333). Additionally, all the raw reads were classified using a naïve Bayesian ribosomal database project classifier for taxonomic assignment at 97% similarity. The mean (with SD) number of OTUs was 412.27 ± 160.87, the minimum number of OTUs was 117, and the maximum number of OTUs was 1013 (Supplementary Table 1).

**Figure 1:**
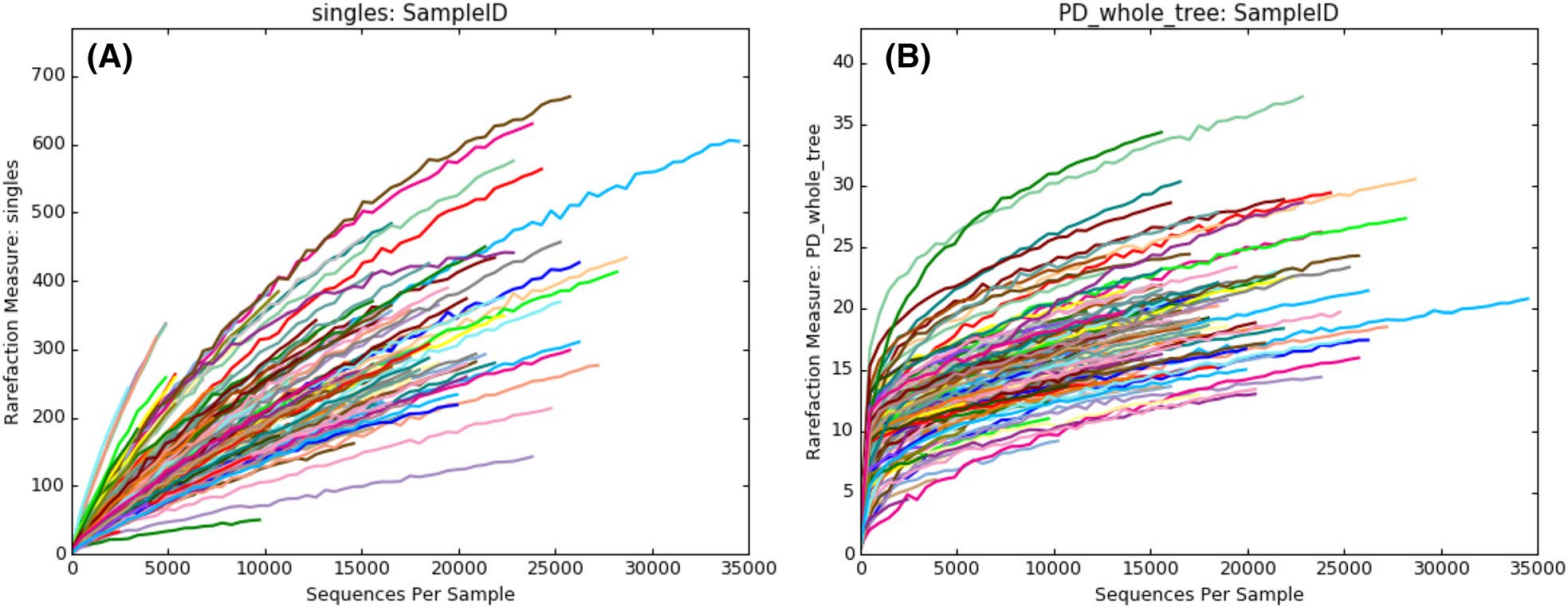
Rarefaction plots of observed species (**A**) and phylogenetic diversity of the whole tree (**B**).

We constructed rarefaction curves to check the sequencing depth^16^ and confirmed that these samples were sequenced deeply enough when viewed as plots of observed species (Fig. 1A) and phylogenetic diversity (PD) of the whole tree (Fig. 1B).

### Alpha diversity of the microbial community

**Figure 2:**
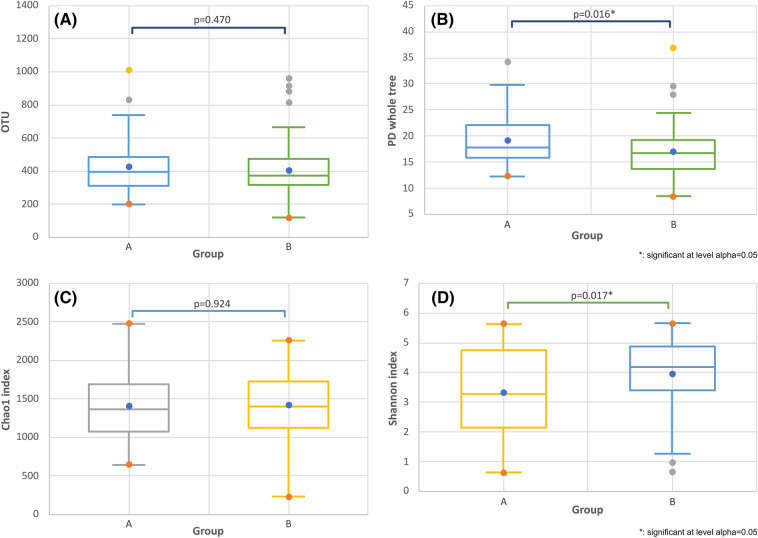
Box plots comparing the two groups in terms of OTUs (**A**), PD of the whole tree (**B**), Chao1 index (**C**), and Shannon index (**D**). *OTU* operational taxonomic unit, *PD* phylogenetic diversity. *Significant at alpha level of 0.05.

There were no statistically significant differences in OTUs between the two groups (*p* = 0.470, Fig. 2A). However, the PD of the whole tree was significantly different in each group (*p* = 0.016, Fig. 2B). In addition, we used Student’s *t*-tests to analyze the alpha diversity of the OS microbiome in each group. The Chao1 index showed that the species richness between the two groups was not significantly different (*p* = 0.924, Fig. 2C). However, the Shannon index showed that there was less diversity among bacterial species in group A than in group B (*p* = 0.017, Fig. 2D).

In addition, we analyzed the alpha diversity of the two groups in terms of controlling variables such as age, gender, and dry eye severity markers (i.e., Schirmer’s test, OSS, TBUT, and OSDI) using a general linear model (GLM). First, we confirmed that the data were normally distributed using the Q–Q plotting method. Therefore, the GLM was used to compare OTUs, PD of the whole tree, the Chao1 index, and the Shannon index in the state that all parameters such as age and dry eye parameters are entered as variables. The OTU (*p* = 0.158) and Chao1 index (*p* = 0.686) were not significantly different. However, for groups A and B, the PD of the whole tree (*p* = 0.002) and the Shannon index (*p* = 0.001) were significantly different.

### Taxonomic composition and dominant genera and phyla

**Figure 3:**
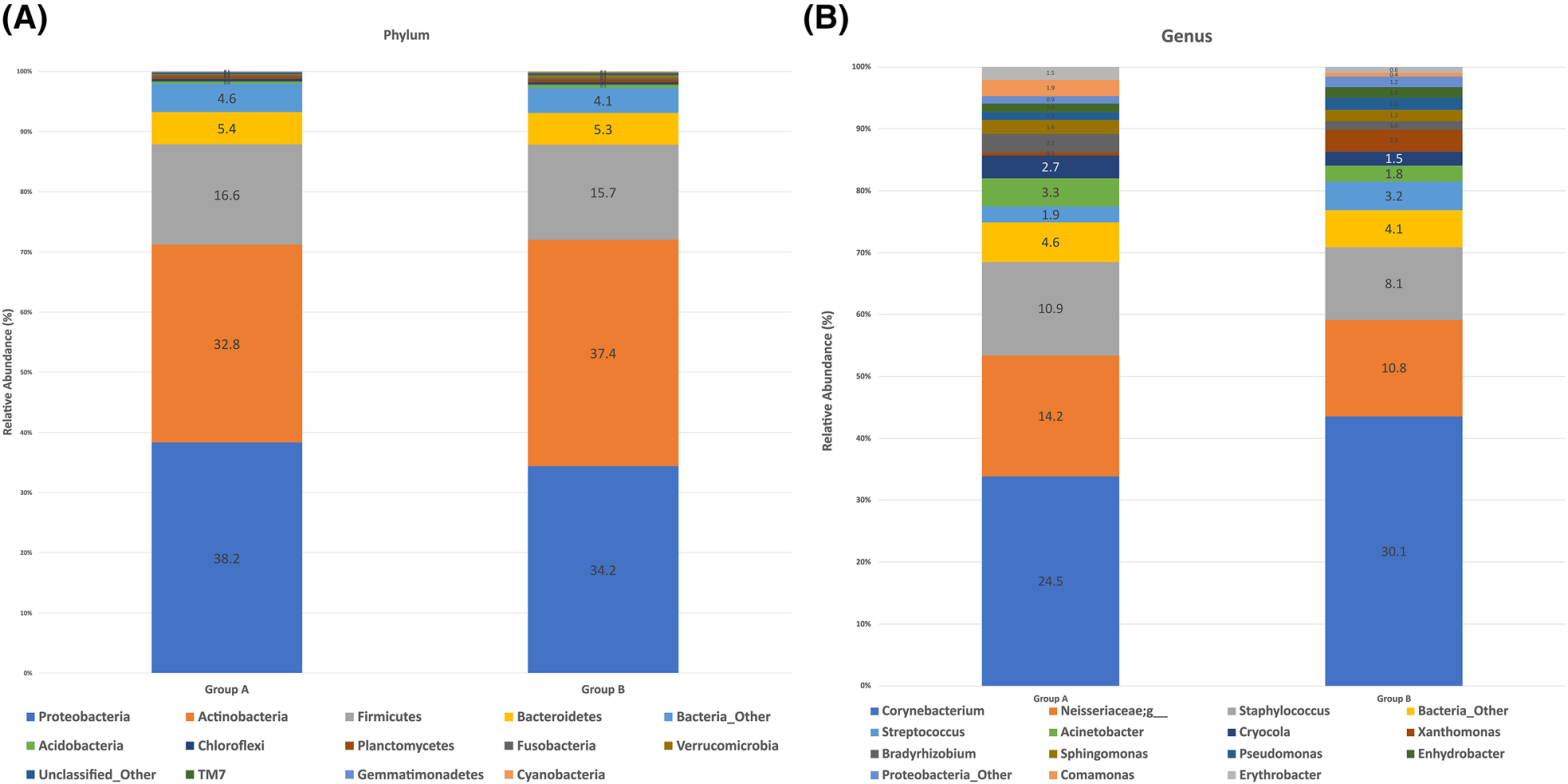
Core microbiome and taxonomic relative abundance of the two groups according to phyla (**A**) and genera (**B**).

16S rRNA gene sequences were classified by phyla and genera to analyze the taxonomic composition of the microbial community. When the swab samples were analyzed, 32 bacterial phyla were detected and these were grouped into 14 major phyla. The most abundant four bacteria (> 1%) were listed for groups A and B. The *Proteobacteria* were most abundant in both groups (means with SD, 38.19% ± 24.72 and 34.24% ± 21.66, respectively), followed by the *Actinobacteria* (32.81% ± 23.06 and 37.43% ± 29.04, respectively), the *Firmicutes* (16.56% ± 18.26 and 15.70% ± 18.28, respectively), and the *Bacteroidetes* (5.39% ± 5.44 and 5.31% ± 5.87, respectively; Fig. 3A). However, there were no statistically significant differences in the relative abundance of these strains between the two groups (Student’s *t*-test, *p* > 0.05).

A total of 698 bacterial genera were detected and these were grouped into 15 major genera. The most abundant 14 bacteria (> 1%) were listed for groups A and B. The *Corynebacteria* were most abundant in both groups (means with SD, 24.54% ± 25.34 and 30.07% ± 31.91, respectively), followed by the *Neisseriaceae* (14.19% ± 24.67 and 10.77% ± 20.76, respectively), the *Staphylococci* (10.91% ± 16.22 and 8.07% ± 16.86, respectively), the *Streptococci* (1.87% ± 4.29 and 3.21% ± 5.18, respectively), the *Acinetobacter* (3.27% ± 5.37 and 1.81% ± 2.70, respectively), the *Cryocola* (2.72% ± 4.73 and 1.54% ± 4.64, respectively), the *Xanthomonads* (0.35% ± 0.62 and 2.46% ± 6.63, respectively), the *Bradyrhizobia* (2.17% ± 4.02 and 0.95% ± 2.95, respectively), the *Sphingomonads* (1.60% ± 2.72 and 1.25% ± 2.22, respectively), the *Pseudomonads* (0.98% ± 2.13 and 1.33% ± 5.05, respectively), the *Enhydrobacter* (0.98% ± 2.38 and 1.17% ± 1.95, respectively), the *Commamonads* (1.94% ± 3.78 and 0.43% ± 1.10, respectively), and the *Erythrobacter* (1.46% ± 3.11 and 0.65% ± 1.19, respectively; Fig. 3B). The *Acinetobacter* were more abundant in group A (Student’s *t*-test was borderline significant, *p* = 0.05), and the *Xanthomonads* were significantly more abundant in group B (Student’s *t*-test, *p* = 0.03).

### Microbial communities on the OS

**Figure 4:**
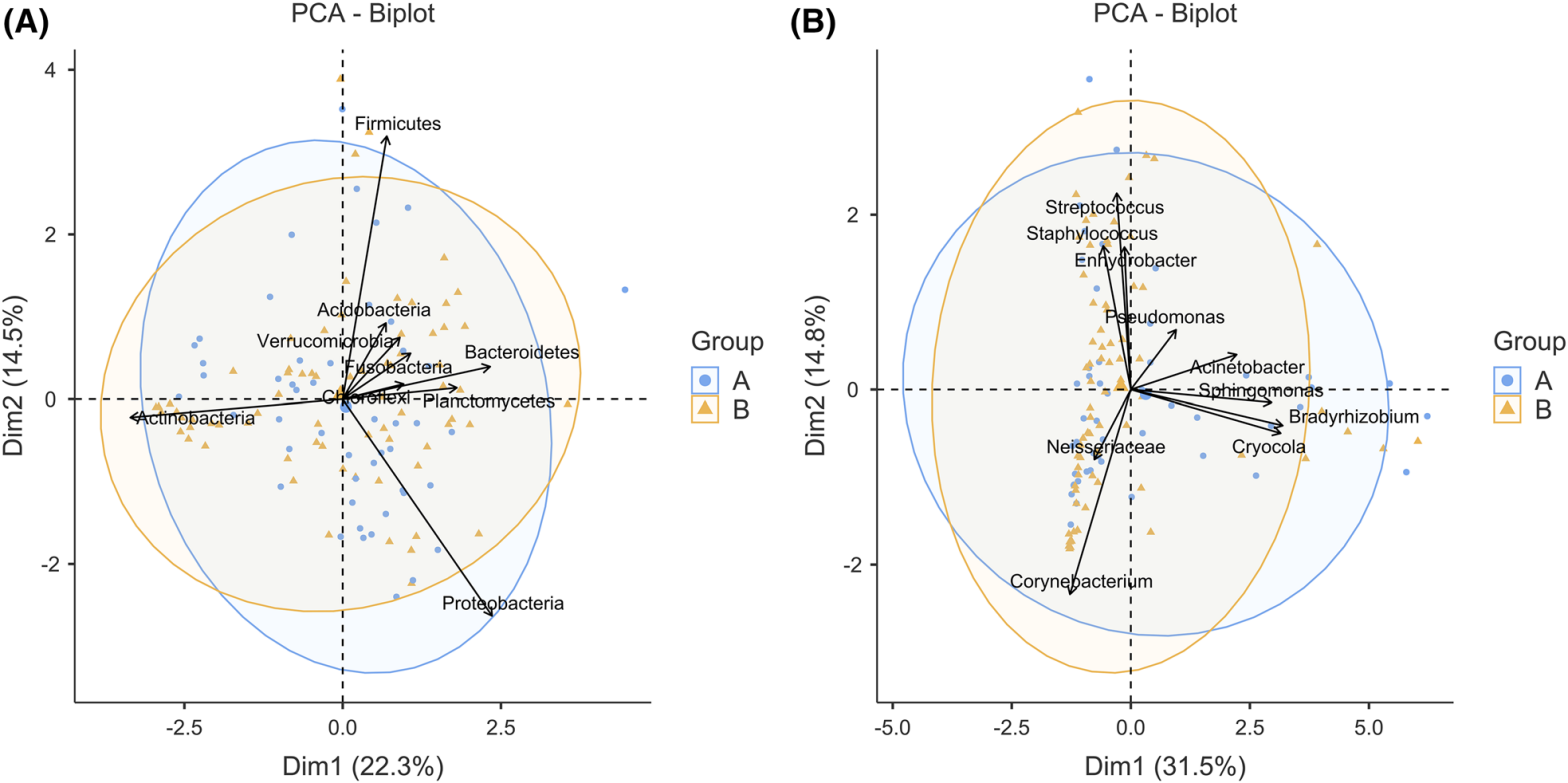
Principle component analysis (PCA) bi-plot showing the beta-diversity of the two groups according to phyla (**A**) and genera (**B**). The numbers on the graphs are the sample IDs of enrolled subjects. *PCA* principle component analysis; *dim* dimension. The two ellipses in each graph represent the 95% confidence ellipse.

Figure 4 shows a principle component analysis (PCA) bi-plot graph of microbial communities at the phylum (Fig. 4A) and genus (Fig. 4B) levels. At both levels, the microbial communities in each group were different but not significantly so. An analysis of similarity (ANOSIM) using the Bray–Curtis method and 9999 permutations found that the microbial communities were not significantly separated at the phylum (*p* = 0.673) or genus (*p* = 0.637) level.

## Discussion

Physiological interactions between the gut microbiota and immune system occur during human development^17^, and gut dysbiosis plays an important role in the pathophysiology of autoimmune diseases such as systemic sclerosis, rheumatoid arthritis, ankylosing spondylitis, and SS^18^. These autoimmune diseases are often associated with ophthalmic disorders such as uveitis and keratoconjunctivitis sicca^19–21^. Keratoconjunctivitis sicca is caused by inflammatory cell infiltration of the lacrimal glands and occurs frequently in patients with SS. Many studies have investigated the association between gut dysbiosis and OS lesions^20,22^. Moon et al. reported that the gut microbiome of patients with SS differed from that of control subjects^22^, and Paiva et al. showed that the severity of SS was inversely correlated with microbial diversity^20^. However, the pathological manifestations of keratoconjunctivitis sicca occur on the OS, and there are fewer microbes on the OS than in the gut. The cornea and conjunctival mucosa are directly exposed to the environment. However, due to defense mechanisms such as antimicrobial proteins in the tears, the protective corneal epithelium, and the presence of various immune cells, few microorganisms flourish on the OS of the uninfected eye^23–25^. Consequently, few studies have analyzed microbial communities on the OS of patients with autoimmune diseases such as SS. Pavia et al. devised a desiccated-stress SS animal model that exhibited significant differences in the microbiota found in the oral mucosa compared with the gut. However, no meaningful differences were observed in conjunctival swab samples because few microbes were present on the OS^20^. Therefore, as demonstrated by our previous research^15^, NGS may be required to analyze these microbiomes.

After controlling for age and dry eye clinical markers using the OSS, TBUT, and Schirmer’s test, we analyzed microbial diversity on the OS of patients with SS (group A) and subjects who did not have SS (group B). The Shannon index was significantly lower in the patients with SS. The Shannon index evaluates microbial diversity by describing the entropy of an ecosystem or in representative samples. Therefore, patients with SS exhibited a decrease in microbial diversity on the OS. In the animal model studied by Pavia et al., the severity of ocular and systemic SS was inversely correlated with gut microbial diversity^20^. Furthermore, Moon et al. found differences in gut microbial diversity between patients with SS and control subjects^22^. In addition, studies of the OS microbiota in subjects with dry eyes, contact lens wearers, and patients with blepharitis or trachoma identified characteristic differences in the abundance of some microbial species^26–29^. Our study is the first to analyze microbial communities on the OS of patients with and subjects without an autoimmune disease. In addition, we tried to exclude subjects who had systemic or ocular diseases, as well as those who had taken systemic agents (e.g., hydroxychloroquine) and topical drugs that could affect the OS microbiota. Finally, we focused on differences in the microbiota that were caused by SS by controlling for age and clinical markers for dry eyes.

We found that the alpha diversity of the microbial community decreased in OS of patients with primary SS. Previous studies reported the presence of a gut–eye lacrimal-gland microbiome axis in patients with SS and that commensal bacteria were involved in protecting the OS and lacrimal glands^20,30^. However, we suggest that protection of the OS may be compromised if the homeostasis of commensal bacteria is disrupted. Previous studies have shown that both Gram-positive and Gram-negative bacteria use quorum sensing to regulate biofilm formation, expression of virulence factors, and motility^31^. Quorum sensing is based on the density of bacterial populations within a particular environment^32^. Therefore, if the homeostasis of commensal bacterial on the OS is disrupted and bacterial diversity decreases, pathogenicity may occur.

In addition, we analyzed species abundance. At the phylum level, *Proteobacteria* and *Actinobacteria* were the predominant microorganisms on the OS, regardless of group. This observation is consistent with previous research^8,15,33,34^. By contrast, *Firmicutes* are generally the predominant microorganisms present in the gut. Moon et al. reported that *Firmicutes* were the predominant microorganisms in the gut of patients with SS^22^. At the genus level, *Corynebacteria* were the predominant microorganisms on the OS. This observation is consistent with previous research. These results suggest that a core community of commensal bacteria are present on the OS and that the species abundance of OS bacteria differs from that of gut bacteria.

Although no statistical significance was found, the clinical meaning of changes in the species abundance of *Proteobacteria* and *Actinobacteria* in the OS of primary SS patients can be found in the following studies. Previous studies have suggested that increased prevalence of *Proteobacteria* is a marker for an unstable microbial community and may be associated with several inflammatory metabolic disorders^35,36^. On the other hand, *Actinobacteria* play important roles in regulating gut permeability, the immune system, metabolism, and the gut–brain axis^37^. *Actinobacteria* can stimulate the production of IL-4 and IL-13, thereby reducing inflammation^38^. In addition, *Actinobacteria* prevent the formation of biofilm and the growth of pathogenic bacteria^39^. The findings related to the OS microbial community of these two phylum may be related to the pathophysiology of the primary SS, and it is thought that additional studies are needed to find a clearer significance. Interestingly, as in our previous study on diabetic patients, genera related to the *Acinetobacter* were abundant on the OS of patients with SS^15^. *Acinetobacter* strains are frequently associated with nosocomial infections, especially in intensive care units, and particular species are reportedly associated with urinary tract infections, burn infections, and hospital acquired pneumonia^40^. *Acinetobacter* colonies on the skin are associated with allergic reactions^41^. In view of these features, the presence of *Acinetobacter* strains on the OS of patients with primary SS, which is an autoimmune disease, may be important. However, the abundance of *Acinetobacter* was low compared to other genera. Although not statistically significant, we found that the abundance of *Corynebacteria* was lower on the OS of patients with SS. Interestingly, *Corynebacterium mastitidis*, which is frequently found on the OS, plays an important role in the local immune response of the corneal and conjunctival mucosa. St Leger et al. found that this organism elicited a commensal-specific IL-17 response from γδ T cells in the ocular mucosa and drove neutrophil recruitment and the release of antimicrobial agents into the tears. In addition, this *Corynebacteria* species was able to protect the OS from infection by pathogenic *Candida albicans* and *Pseudomonas aeruginosa*^42^. Further research will be needed to understand how these observations may be linked and whether ocular commensal bacteria can train cells involved in the immune response to resist pathogens.

Our study had some limitations, and our results should be interpreted with caution due to the limited sample size and the paucity of bacteria present on the OS. Although differences in the bacterial populations were observed in the PCA bi-plots at the phylum and genus levels, significant differences in the Bray–Curtis distances were not found. In addition, there are practical limitations inherent to analysis of bacterial species on the OS using DNA sequencing. However, we were able to exclude the possibility of contamination of our sterile cotton swab samples with bacterial DNA. In addition, the proportions of microorganisms that we identified on the OS were similar to those described by previous studies. Although 16S rRNA sequencing with sparse bacteria from the OS may generate some false positive reads and sequencing errors, NGS has many advantages over conventional culture methods^34,43^. This is the first study to investigate the microbiota of the OS in patients with SS using NGS, while controlling for dry eye clinical markers.

In conclusion, SS decreases the diversity of the microbial community on the OS of patients and affects the abundance of particular bacterial strains at the phylum and genus levels. It is unclear whether these changes result from or cause immunological disorders, but these possibilities may be investigated in future studies to understand more fully the pathophysiology of SS.

## Materials and methods

### Study design and subjects

This prospective case–control study was conducted in accordance with the Declaration of Helsinki, and the study protocol was approved by the Institutional Review Board of Incheon St. Mary’s Hospital (assignment no. OC15SISI0010). In addition, written informed consent was obtained from all participants. Patients were recruited at the Cornea Service of Incheon St. Mary’s Hospital. Patients who wore contact lenses or had any of the following conditions or treatments that could affect eye dryness were excluded: meibomian gland dysfunction above grade 2^44^, allergic conjunctivitis, ocular surgery within the last 6 months, punctal plug insertion, or topical treatments other than artificial tears in the 3 months before this study. Patients who were taking medication for a systemic disease such as autoimmune diseases, diabetes, hypertension, thyroid disease, allergic disease, or a depressive disorder were excluded.

G* power software (latest ver. 3.1.9.7; Heinrich-Heine-Universität Düsseldorf, Düsseldorf, Germany) was used to calculate the number of study subjects. Effect size was set to 0.5, alpha error was 0.05, and power (1-beta error) was set to 0.80. Since the number of SS patients was expected to be smaller than that of the control group, the allocation ratio was set to 1.5. According to this calculation result, at least 42 SS patients and at least 64 subjects as control had to be recruited. A total of 120 subjects were included in this study. In total, 15 subjects were male and 105 subjects were female. All of the study subjects had been referred to ophthalmology departments to investigate a diagnosis of primary SS and reported having dry eyes (e.g., dryness, itching, or the sensation of a foreign body) and a dry mouth (e.g., frequent thirst, dry feeling in the mouth and throat, burning or tingling sensation in the mouth and tongue, or cracked lips). All subjects had symptoms for ≥ 3 months, low TBUT (≤ 7 s), and low Schirmer I test without anesthesia value (< 10 mm/5 min). We used Sjögren’s International Collaborative Clinical Alliance (SICCA) classification criteria announced in 2012 to diagnose SS^45^. In total, 48 patients (group A) were diagnosed with primary SS and the remaining 72 subjects (group B) were classified as having dry eyes and dry mouth symptoms that were unrelated to SS. Patients who had at least two of the following three characteristics were diagnosed as having primary SS: positive serum anti-SSA/Ro and/or anti-SSB/La or [positive rheumatoid factor and antinuclear antibodies titer ≥ 1:320], labial salivary gland biopsy exhibiting focal lymphocytic sialadenitis with a focus score ≥ focus/4 mm^2^, and keratoconjunctivitis sicca with OSS ≥ 3.

### Assessment of dry eye clinical parameters

OSS that included corneal and conjunctival staining scores, Schirmer’s test, and TBUT were evaluated by the same ophthalmologist (HBH). Dry eye symptoms were graded numerically from 0 to 4 using the OSDI, and the sum of these scores was used in the analyses. The OSDI, developed by the Outcomes Research Group at Allergan (Irvine, CA, USA), is a 12-item questionnaire that assesses vision-related functioning. Each item is scored on a 5-point scale, resulting in a total OSDI score ranging from 0 (no symptoms) to 100 (maximal symptoms)^46^. To measure TBUT, fluorescein was instilled from a fluorescein strip in a drop of saline (Haag-Streit, Koeniz, Switzerland). The fluorescein was applied to the superior-temporal bulbar conjunctiva with participants instructed to gaze infero-nasally, and the TBUT was measured using a slit-lamp under a cobalt blue light^47^. The mean value of three TBUT measurements was used in this study. The OSS was measured in accordance with the SICCA registry ocular examination protocol^48^. Corneal punctate epithelial erosions (PEEs) were evaluated and scored after staining with a fluorescent dye. Corneal scores were assigned as follows: PEEs absent = 0 points; one to five PEEs = 1 point; six to 30 PEEs = 2 points; and more than 30 PEEs = 3 points. An additional point was assigned if more than one patch of confluent aggregated staining was found in the pupillary area or if filaments present on the cornea were stained. The maximum possible score for each cornea was 6 points. To assess conjunctival staining scores, fluorescein was washed out with non-preserved saline solution, after which 1% lissamine green dye (Leiter’s Pharmacy, San Jose, CA, USA) was applied to the inferior conjunctival fornix. After several blinks, conjunctival staining scores in the temporal and nasal bulbar conjunctiva were evaluated separately as follows: up to nine dots = 0 points; 10 to 32 dots = 1 point; 33 to 100 dots = 2 points; and more than 100 dots = 3 points. The maximum possible score for the conjunctiva (i.e., temporal and nasal regions) was 6 points. For Schirmer’s test, standard Schirmer strips (Eagle Vision, Memphis, TN, USA) were placed in the lateral one-third of the lower eyelid, without topical anesthesia. After 5 min, the length of the wet portion of the strip was measured.

### Sample collection and DNA extractions

The sampling and DNA extraction procedures used in this study were the same as those used in our previous research^15^. In the absence of topical anesthesia, sterile dry cotton swabs (MEDIUS Corp., Tokyo, Japan) were used to sample four different locations: left upper bulbar conjunctiva, left lower bulbar conjunctiva, right upper bulbar conjunctiva, and the right lower bulbar conjunctiva. Since the bacterial burden of OS is relatively low, in order to obtain as much burden as possible, samples were collected after swabbing four conjunctival spaces by maximizing the contact surface using one sterile cotton swab per subject. Each cotton swab was placed into a 1.5 mL sterilized Eppendorf microtube and frozen at – 70 °C until DNA extraction. Nucleic acids were extracted from the swabs using an i-genomic Soil DNA Extraction Mini Kit (iNtRON, Seoul, South Korea) with a bead-beating apparatus, in accordance with the manufacturer’s instructions with the following method alterations. To enhance cell lysis, approximately 1.5 × lysis buffer was added to the samples for 30 min. DNA concentrations were measured using a Qubit fluorometer (Invitrogen, Gaithersburg, MD, USA).

### 16S rRNA gene library preparation

The hypervariable region 4 (V4) of the 16S rRNA gene was amplified from the extracted DNA using the 515F (5ʹ–GTGYCAGCMGCCGCGGTAA–3ʹ) and 806R (5ʹ–GGACTACNVGGGTWTCTAAT–3ʹ) primers^49^. The polymerase chain reaction (PCR) conditions were 94 °C for 3 min, followed by 35 cycles of 94 °C for 45 s, 50 °C for 60 s, and 72 °C for 90 s, followed by a final extension step at 72 °C for 10 min. Each sample was amplified in three replicate 25-µL PCR reactions, purified using the UltraClean PCR clean-up kit (Mo Bio Laboratories, Solana Beach, CA, USA), and combined into a single tube. Purified DNA was quantified by incorporating Picogreen (Invitrogen), in accordance with the manufacturer’s instructions.

### Sequencing data analyses

Libraries for the V4 region alone were generated, and 16S rRNA-based MiSeq sequencing was conducted using standard Earth Microbiome Project protocols (http://www.earthmicrobiome.org/emp-standardprotocols/) at the Argonne National Laboratory (Lemont, IL, USA). QIIME version 1.9.1 was used for processing all sequencing reads^50^. Sequences were de-multiplexed according to their barcodes, merged, and quality-filtered using the default parameters. OTUs were clustered using the latest Greengenes 13_8 reference sequences at 97% similarity (approximately corresponding to species-level OTUs), using the UCLUST algorithm. To remove chimera sequences, UCHIME within USEARCH was used^51^. Then, sequences were aligned using PyNAST software (version 1.2, Boulder, USA)^52^. Representative OTU sequences were evaluated taxonomically using the ribosomal database project classifier^53^, retrained on the Greengenes database (13_8)^54^, and mitochondrial sequences were filtered out of the OTU table. OTU reads found in negative control samples were removed from the OTU table.

Unlike the gut microbiome, the OS microbiome does not have much burden, so the difference in sequencing depth between samples is relatively large. Therefore, we transformed our data to proportions by dividing the reads for each OTU in a sample by the total number of reads in that sample for normalizing data. This method is known as total sum normalization (TSS) method^55^. To calculate species diversity and richness, alpha diversity analyses using the Shannon and Chao1 indexes were processed using the QIIME script. To measure the similarity among communities, beta diversity was calculated and PCA bi-plots were constructed.

### Negative controls

All experimental procedures including sampling, DNA extraction, preparation, and sequencing were implemented in a consistent manner. DNA extraction and PCR amplification of samples from eyes and unused sterile cotton swabs were all performed using exactly the same procedures. When DNA was extracted from the unused sterile cotton swabs, the quantity of DNA produced was very low (i.e., less than 0.05 ng DNA µL^−1^), and no DNA was amplified by the bacteria-specific primers (i.e., 27F and 1494R did not generate a PCR band). To minimize variability from any batch effects during different sequencing runs, all samples were sequenced using the same sequencing run. Negative controls were generated in reaction mixtures containing no template. During the sequence data analyses, OTU reads identified in DNA extractions of negative controls were removed from the sequencing data using metadata-based filtering.

### Diagnostic testing procedure

The testing procedure for ophthalmic examinations was as follows:

1. Subjective interview regarding symptoms of dry eyes (i.e., OSDI) and recording the patient’s medical history (HBH and KDK).
2. Slit-lamp evaluation of the cornea, conjunctiva, eyelids, and Meibomian glands (HBH).
3. Schirmer’s test (without topical anesthesia; HBH).
4. TBUT test using fluorescein dye, repeated 3 times (HBH).
5. Fluorescein and lissamine green staining of the cornea and conjunctiva (HBH).

Thereafter, subjects returned after 2 weeks and were classified into two groups according to whether the rheumatologist confirmed a diagnosis of SS.

6. Conjunctival swab sampling (HBH, KDK and JMY).
7. DNA extraction and sequencing analysis (BN).

### Statistical analyses

All data are expressed as means ± SD. Statistical analyses were performed using JAMOVI software (version 1.6; retrieved from http://www.jamovi.org) and XLSTAT statistical software (version 2021.3.01; Addinsoft SARL, Paris, France, http://www.xlstat.com/en/company/). Group differences in age and dry eye parameters were evaluated using Student’s *t*-test after confirming that the data were normally distributed using the Kolmogorov–Smirnov test (p > 0.05). Group differences in alpha diversity (i.e., Shannon and Chao1 indexes) were analyzed using a GLM by controlling for age and dry eye parameters as covariates. Before performing GLM and PCA, we checked the linearity of the plot using the Q-Q plotting method and checked the normal distribution of the data in advance. PCA bi-plot was used to create a visual representation of the data, with enrolled subjects and the various microbial strains arranged into two groups. To compare the microbial communities associated with the two groups, an ANOSIM was performed using Bray–Curtis dissimilarities after 9999 permutations and the “vegan” package in ANOSIM. We used Student’s *t*-test to compare species richness between the groups. A *p*-value < 0.05 was considered statistically significant.

## Supplementary Information

Supplementary Table 1.

## Data Availability

We declare that all experimental data are described accurately in this manuscript. For further information, please contact the corresponding author.
