# Supplementary material for: Bacterial distribution on the ocular surface of patients with primary Sjögren’s syndrome
Source: Sci Rep. 2022 Feb 2;12:1715. doi: 10.1038/s41598-022-05625-w (PMC8810764; doi:10.1038/s41598-022-05625-w)
Supplement: Supplementary file 1 — Supplementary Table 1. [file 41598_2022_5625_MOESM1_ESM.docx]

**Supplementary table 1. Sequencing results for collected samples**

| **Number of samples** | | | | | 120 | | | | |  |  |  |  |
| --- | --- | --- | --- | --- | --- | --- | --- | --- | --- | --- | --- | --- | --- |
| **Number of observations** | | | | | 10668 | | | | |  |  |  |  |
| **Total count** | | | | | 1890438 | | | | |  |  |  |  |
| **Counts/sample summary** | | | | | | | | | |  |  |  |  |
| **Min** | | | | | 5001 | | | | |  |  |  |  |
| **Max** | | | | | 37333 | | | | |  |  |  |  |
| **Median** | | | | | 15928 | | | | |  |  |  |  |
| **Mean** | | | | | 15753.7 | | | | |  |  |  |  |
| **Std. dev.** | | | | | 6354.1 | | | | |  |  |  |  |
| **Sample name / Counts / OTU** | | |  |  |  |  |  |  |  |  |  |  |  |
| **HE.1** | 9338 | 216 | | ***HE.128*** | 10834 | 314 | **HE.39** | 20938 | 364 | | ***HE.69*** | 21372 | 432 |
| **HE.10** | 14886 | 253 | | ***HE.129*** | 17638 | 476 | **HE.40** | 17907 | 351 | | **HE.7** | 25409 | 469 |
| ***HE.101*** | 10500 | 506 | | ***HE.13*** | 18740 | 373 | ***HE.41*** | 7183 | 191 | | ***HE.70*** | 28362 | 588 |
| **HE.102** | 11270 | 314 | | ***HE.130*** | 16665 | 487 | ***HE.42*** | 6350 | 253 | | **HE.71** | 27491 | 374 |
| ***HE.103*** | 9127 | 343 | | ***HE.131*** | 28680 | 666 | **HE.43** | 10186 | 322 | | **HE.72** | 21716 | 415 |
| ***HE.104*** | 5044 | 467 | | ***HE.132*** | 23235 | 917 | **HE.44** | 10110 | 337 | | **HE.73** | 8779 | 331 |
| **HE.105** | 8996 | 478 | | ***HE.133*** | 19070 | 479 | **HE.45** | 8654 | 382 | | **HE.74** | 12983 | 426 |
| **HE.106** | 15199 | 624 | | ***HE.134*** | 19444 | 415 | **HE.46** | 5668 | 245 | | ***HE.75*** | 24078 | 201 |
| **HE.107** | 24284 | 958 | | ***HE.14*** | 16067 | 299 | **HE.47** | 17802 | 326 | | ***HE.76*** | 19092 | 376 |
| **HE.108** | 37333 | 883 | | **HE.16** | 5001 | 463 | **HE.48** | 15109 | 287 | | ***HE.77*** | 11232 | 262 |
| **HE.11** | 11071 | 343 | | **HE.17** | 5999 | 132 | **HE.49** | 26247 | 390 | | ***HE.79*** | 18090 | 430 |
| **HE.110** | 12554 | 338 | | ***HE.19*** | 6889 | 347 | **HE.50** | 26342 | 450 | | **HE.8** | 20535 | 336 |
| ***HE.111*** | 14098 | 441 | | ***HE.20*** | 13698 | 382 | **HE.51** | 15712 | 281 | | ***HE.80*** | 16217 | 354 |
| **HE.112** | 13418 | 332 | | **HE.21** | 20072 | 308 | **HE.52** | 5647 | 262 | | **HE.81** | 10788 | 521 |
| ***HE.113*** | 22253 | 399 | | **HE.22** | 17061 | 388 | **HE.53** | 12374 | 278 | | **HE.82** | 8660 | 226 |
| ***HE.114*** | 20467 | 478 | | ***HE.23*** | 5665 | 278 | **HE.54** | 11877 | 273 | | ***HE.83*** | 17969 | 441 |
| **HE.115** | 15704 | 359 | | **HE.25** | 18070 | 315 | ***HE.55*** | 15729 | 444 | | ***HE.84*** | 16584 | 741 |
| **HE.116** | 15777 | 387 | | **HE.26** | 18756 | 376 | **HE.56** | 16381 | 547 | | ***HE.85*** | 22066 | 674 |
| ***HE.117*** | 24461 | 813 | | ***HE.27*** | 12697 | 466 | **HE.57** | 6399 | 233 | | ***HE.86*** | 18383 | 443 |
| **HE.118** | 20144 | 346 | | ***HE.28*** | 10822 | 254 | **HE.58** | 12726 | 452 | | ***HE.88*** | 5459 | 355 |
| **HE.119** | 21053 | 507 | | ***HE.29*** | 16813 | 397 | **HE.6** | 5570 | 355 | | **HE.89** | 26479 | 546 |
| **HE.12** | 9722 | 232 | | **HE.3** | 7648 | 289 | **HE.60** | 17907 | 448 | | **HE.9** | 18060 | 346 |
| **HE.120** | 21610 | 608 | | ***HE.30*** | 18695 | 401 | **HE.61** | 14328 | 365 | | **HE.90** | 14304 | 285 |
| **HE.121** | 20859 | 326 | | **HE.31** | 9024 | 227 | **HE.62** | 11692 | 407 | | ***HE.91*** | 15788 | 597 |
| **HE.122** | 22837 | 476 | | **HE.32** | 7547 | 320 | ***HE.63*** | 9641 | 258 | | **HE.92** | 22866 | 830 |
| ***HE.123*** | 22466 | 527 | | **HE.33** | 9773 | 117 | **HE.64** | 13689 | 389 | | ***HE.95*** | 16665 | 313 |
| **HE.124** | 19885 | 551 | | **HE.34** | 15727 | 381 | **HE.65** | 14736 | 295 | | ***HE.96*** | 18737 | 605 |
| ***HE.125*** | 17911 | 319 | | **HE.36** | 11523 | 340 | **HE.66** | 16369 | 511 | | ***HE.97*** | 26113 | 1013 |
| ***HE.126*** | 16839 | 480 | | **HE.37** | 24870 | 293 | **HE.67** | 6186 | 249 | | ***HE.98*** | 25294 | 588 |
| ***HE.127*** | 17472 | 415 | | **HE.38** | 15617 | 539 | **HE.68** | 17261 | 475 | | **HE.99** | 5229 | 376 |

Italics and underlined sample IDs indicate primary SS patients.
